# Bulked Segregant Analysis Revealed the Common Resistant QTLs Associated with Fusarium Ear Rot and Gibberella Ear Rot in Maize

**DOI:** 10.3390/plants15091401

**Published:** 2026-05-04

**Authors:** Haiyan Zhang, Weili Cai, Wenyi Li, Luyao Duan, Zhenyu Zhang, Chengjia Zou, Ling Li, Lin Li, Runtian Xiao, Lina Cui, Xiao Li

**Affiliations:** 1 Institute of plant protection, Sichuan Academy of Agricultural Sciences, Chengdu 610066, China; zhanghaiyan@scsaas.cn (H.Z.); caiwl2024@scaas.cn (W.C.); liwenyi@scsaas.cn (W.L.); duanluyao@scsaas.cn (L.D.); zhangzhenyu@scsaas.cn (Z.Z.); zouchengjia@scsaas.cn (C.Z.); ll3114@nwafu.edu.cn (L.L.); luojinyunlilin@163.com (L.L.); 17381469596@163.com (R.X.); 2 Key Laboratory of Integrated Crop Pest Management in Southwest China, Ministry of Agriculture, Chengdu 610066, China

**Keywords:** disease resistance, fusarium ear rot, Gibberella ear rot, quantitative trait loci, maize

## Abstract

Maize ear rot, primarily caused by *Fusarium verticillioides* (Fusarium ear rot, FER) and *Fusarium graminearum* (Gibberella ear rot, GER), is a devastating disease that causes significant yield losses and mycotoxin contamination. Breeding resistant varieties is the most effective control strategy, but this requires the identification of stable genetic loci for resistance. In this study, we employed bulked segregant analysis (BSA) on two F_2_ mapping populations to identify quantitative trait loci (QTLs) conferring resistance to FER and GER. We identified five and eleven QTLs for FER and GER, respectively. Notably, chromosome 4 was identified as a major hotspot for resistance to both diseases, and there was a co-localization of the FER QTL (*qFER4.05*) and GER QTL (*qGER4.05-1*) within a 58.58–71.34 Mb interval on bin 4.05, suggesting a potential locus for broad-spectrum resistance. Within this overlapping region, we identified 18 high-confidence candidate genes, including genes encoding leucine-rich repeat receptor-like kinases (LRR-RLKs), remorin, cytochrome P450 monooxygenases, and wall-associated receptor kinase-like (WAKL) protein, all with established roles in plant defense. These findings advance the understanding of the genetic architecture of ear rot resistance and provide critical resources for marker-assisted breeding to develop maize hybrids with durable resistance to both FER and GER.

## 1. Introduction

Maize ear rot, one of the most devastating diseases in global maize production, not only reduces the yield and quality of harvested maize but also is fatal to humans and animals that consume the contaminated grain, which contains mycotoxins from the pathogen *Fusarium* spp. [1,2]. *Fusarium verticillioides* and *Fusarium graminearum* are the two most important pathogens that can cause Fusarium ear rot (FER) and Gibberella ear rot (GER), respectively [3,4]. *Fusarium* spp. can survive in plant residue, healthy seeds, and soil, and exhibits a high level of genetic diversity, which allows them to easily adapt to a variety of environmental changes and fungicides, resulting in chemical and agronomic measures usually being ineffective [2,5,6]. The best strategy to prevent and control maize ear rot is breeding and promoting resistant varieties, which requires us to understand the genetics of resistance clearly and identify the alleles that can significantly reduce damage caused by *F. verticillioides* and *F. graminearum* [7,8]. Molecular marker-assisted selection and polyresistant polymerization, which depend on functional genes and stable quantitative trait loci (QTLs), are effective ways to improve the efficiency of breeding [9]. It is very important to identify novel resistance genes and QTLs against the two pathogens in order to develop a sustainable solution to FER and GER problems in maize production.

Resistance to ear rot in maize is a complex trait that is controlled by many genes with minor effects, and it is strongly influenced by environmental conditions [10,11,12,13]. As of yet, numerous studies have attempted to identify stable inheritance of resistance loci for FER and GER.

For FER resistance, there have been relatively more research efforts focused on the detection of stable QTLs and the identification of resistance genes compared to GER resistance. Several studies have identified QTL associated with FER using cross-populations, such as F_2_, F_2:3_, and RILs. Chen et al. discovered a stable and significant resistance effect QTL (bin 4.05/4.06) for FER resistance affecting approximately 17.95% of the phenotypic variation using 210 F_2:3_ families, and accounting for up to 35.2% of the phenotypic effect in near-isogenic lines (NILs) when in homozygosity [14]. Maschietto et al. detected three significant resistance QTLs (bin 6.01, bin 7.02, and bin 9.05) for FER resistance using 188 F_2:3_ families, explaining 15.45–19.47% of phenotypic variation [15]. In two additional studies based on RIL populations, Ding et al. detected two stable QTLs (bin3.04) across multiple environments by using 187 RILs, explaining 13–22% of the phenotypic variation [16], and Li et al. detected a resistance QTL (bin 4.06) with 10.2% of the phenotypic variation by using 250 RILs [17]. Certainly, many other studies on FER resistance use linkage mapping, a widely applied method that produces fewer false-positive results and offsets the low allelic diversity in progeny populations [18,19,20]. In addition, work on GWAS for FER has been performed by many other studies, such as [21,22,23,24,25] and others. However, no genes have been isolated by map-based cloning to date, and few stable QTLs have been verified for molecular breeding.

For GER resistance, some progress has been made in the detection of stable QTLs and the identification of resistance genes [26]. Three QTLs for resistance to GER were detected in different environments by using 144 recombinant inbred lines (RILs) [10]. Another study identified one QTL associated with GER in multiple environments by using 410 RILs [27]. Moreover, the important loci of qRger7.1 and qRger7.2 have been identified, which conferred resistance to GER by using two F_2_ populations [28]. Five stable QTLs were detected by 204 RILs, and the largest effect QTL (qGER4.09) has been validated using 588 F_2_ individuals [29]. Recently, two environment-specific resistant QTLs (qGER3.2 and qGER3.4), which had a PVE greater than 10%, were detected using 246 double-haploid inbred lines [30]. Moreover, work on GWAS for GER has been performed by many other studies. Eight QTLs were detected, jointly explaining 34% of the genetic variance by using 500 double-haploid lines derived from two European maize landraces [31]. Ten significantly associated SNPs linked to GER resistance, with the greatest phenotypic variation explained (PVE) value at 9.07%, were detected by a genome-wide association study using 316 diverse inbred lines [32]. Additionally, sixty-nine quantitative trait nucleotides conferring resistance to GER were detected using 344 inbred lines [33]. Although several QTLs or SNPs for resistance to GER have been detected using different populations, few underlying genes have been identified because of the different populations and environments.

Despite extensive research on FER and GER resistance, several critical gaps remain unresolved. First, most previous studies have investigated FER and GER resistance independently, using different populations and experimental conditions, making direct comparison of their genetic architectures difficult. Second, the identification of common genetic loci conferring resistance to both diseases has been limited. Third, the resolution of QTL intervals from bulked segregant analysis (BSA) studies has often been insufficient for precise candidate gene identification. These limitations hinder the development of broad-spectrum resistance breeding strategies.

This study employed BSA to identify QTLs conferring resistance to both FER and GER in maize. Two F_2_ populations, derived from resistant × susceptible crosses, were phenotyped under the same environmental conditions. We hypothesize that simultaneous analysis of FER and GER under comparable environmental conditions will reveal shared genetic architecture, and the identification of co-localized QTLs on chromosome 4 will provide targets for developing maize varieties with durable, broad-spectrum resistance to both ear rot diseases. We present the first simultaneous BSA mapping study of FER and GER resistance under comparable conditions, enabling direct comparison of their genetic architectures, and identified chromosome 4 as a major hotspot for both diseases and discovered co-localized QTLs (*qFER4.05* and *qGER4.05-1*) within a 58.58–71.34 Mb interval, suggesting potential broad-spectrum resistance loci. We further provided detailed candidate gene analysis within the overlapping region, prioritizing genes directly involved in plant immunity and discussing the potential of these loci for application in marker-assisted breeding to enhance FER and GER resistance.

## 2. Results

### 2.1. Phenotype of the FER and GER Resistance

**Figure 1:**
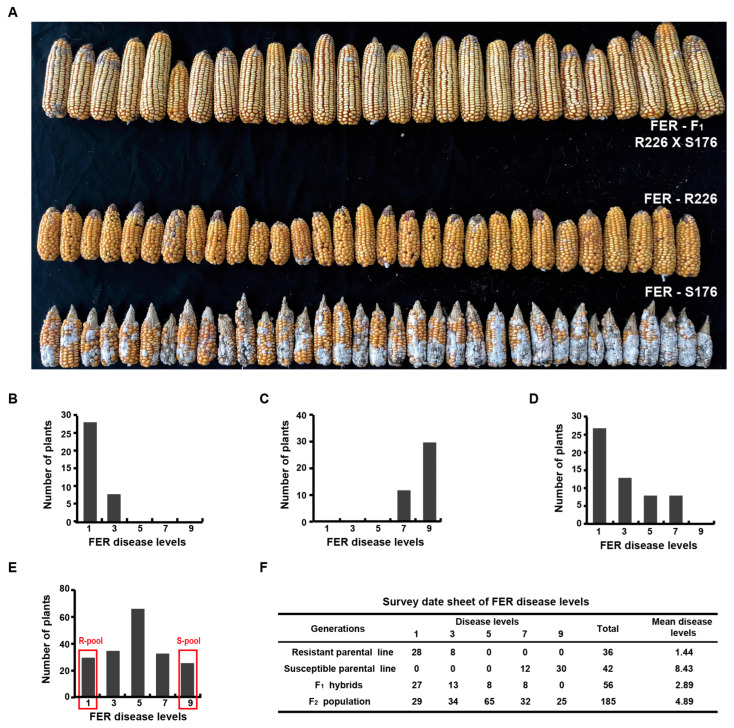
Phenotypic diversity and frequency distribution of resistance to Fusarium ear rot (FER) at harvest. Phenotypic diversity of resistance to FER for the two parental inbred lines and their F_1_ hybrids (**A**), frequency distribution of resistance to FER for the resistant parental inbred line (**B**), the susceptible parental inbred line (**C**), the F_1_ hybrids (**D**), and the F_2_ population (**E**), respectively, and the phenotypic survey results for the whole generations of the QTL mapping population to FER resistance (**F**).

The frequency distribution of FER resistance following artificial inoculation with *F. verticillioides* is presented in Figure 1A–F. The resistant parental inbred line, R226, exhibited high resistance to FER (mean disease level at 1.44) (Figure 1A,B,F), whereas the susceptible parental inbred line, S176, was highly susceptible (mean disease level at 8.43) (Figure 1A,C,F). The majority of F_1_ hybrids (71.43%) were resistant, with a mean disease level of 2.89 (Figure 1A,D,F), suggesting that the major FER resistance alleles are likely dominant. The F_2_ population displayed a continuous variation in disease severity (Figure 1E,F) with a mean disease level of 4.89. This segregation pattern is consistent with quantitative inheritance, suggesting that FER resistance in R226 is a polygenic trait.

**Figure 2:**
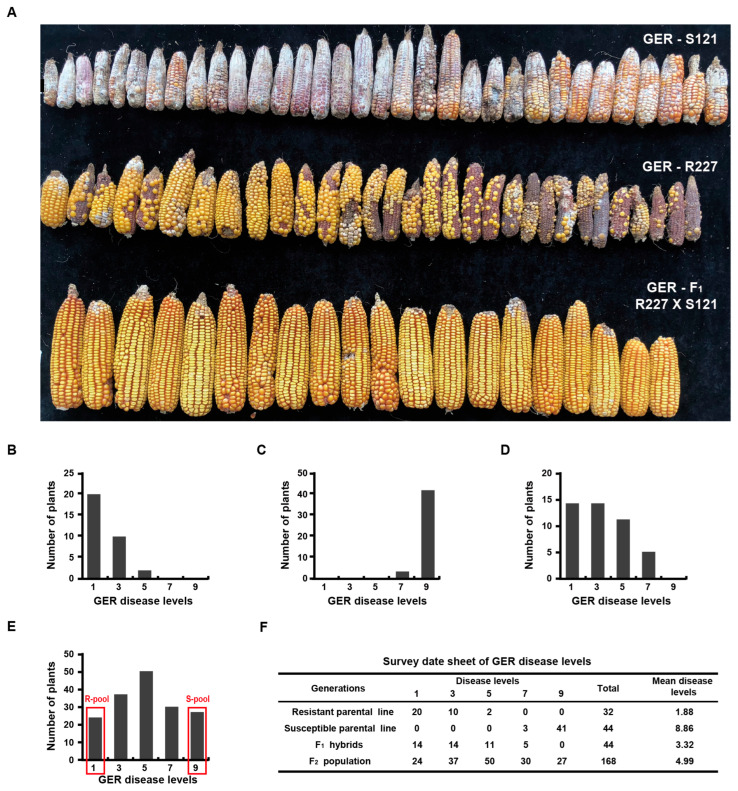
Phenotypic diversity and frequency distribution of resistance to Gibberella ear rot (GER) at harvest. Phenotypic diversity of resistance to GER for the two parental inbred lines and their F_1_ hybrids (**A**), frequency distribution of resistance to GER for the resistant parental inbred line (**B**), the susceptible parental inbred line (**C**), the F_1_ hybrids (**D**), and the F_2_ population (**E**), respectively, and the phenotypic survey results for the whole generations of the QTL mapping population to GER resistance (**F**).

Similarly, the distribution of GER resistance after inoculation with *F. graminearum* is shown in Figure 2A–F. The resistant parental, inbred line R227, showed strong resistance (mean disease level at 1.88) (Figure 2A,B,F), while the susceptible parental inbred line S121 showed severe symptoms (mean disease level at 8.86) (Figure 2A,C,F). A significant proportion of F_1_ hybrids (63.64%) were resistant, with a mean disease level of 3.32 (Figure 2A,D,F). The F_2_ population also exhibited a continuous distribution of GER symptoms (Figure 2E,F) with a mean disease level of 4.99, indicating that the resistance to GER in the resistant parental inbred line R227 is also quantitatively inherited.

### 2.2. BSA Mapping of FER Resistance

BSA was performed using two pooled DNA samples representing extreme phenotypes from the F_2_ population (derived from the cross between R226 and S176). Whole-genome sequencing of the susceptible and resistant bulks yielded 185,305,948 and 187,834,729 clean reads, corresponding to 55.51 and 56.27 Gb of high-quality sequence (Q30 ≥ 93.42%), respectively. The clean reads were aligned to the reference genome, achieving mapping rates of 97.42% for the susceptible bulk and 98.58% for the resistant bulk (Table S1). A total of 10,714,248 SNPs were identified (Table S2). A total of 2,857,322 high-quality SNPs were identified and used for association analysis between the two pools after filtering diversity loci (Table S2).

**Figure 3:**
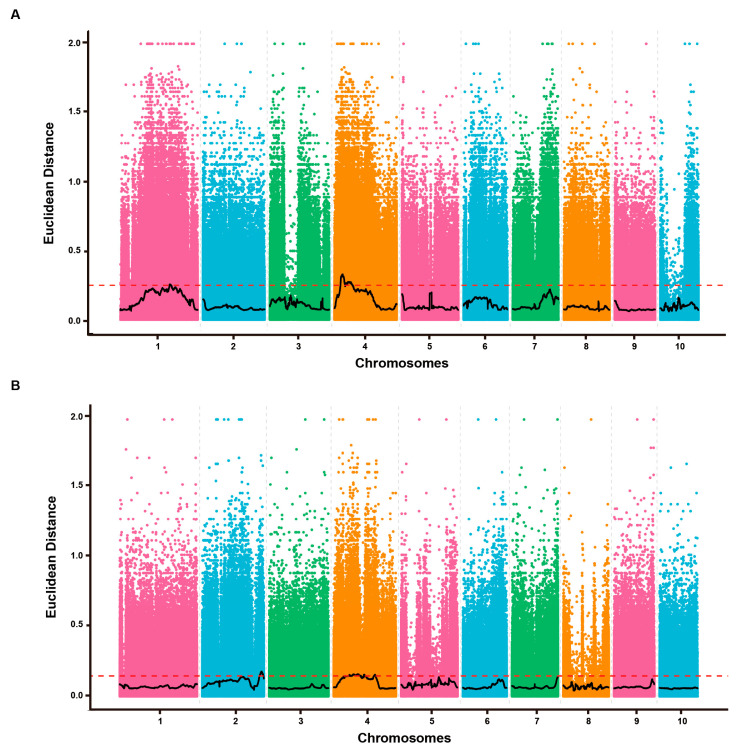
The distribution of ED_values for high-quality SNPs to the FER (**A**) and GER (**B**) resistance on all chromosomes. The ED value represents the size of the difference between the FER_S and FER_R DAN pools. The red dashed line represents the correlation threshold, which was calculated as 0.25.

The Euclidean distance (ED) value for each high-quality SNP was calculated to assess the association between allele frequency and FER resistance in the contrasting bulks (FER_S and FER_R). The ED_values were plotted across all maize chromosomes (Figure 3A). A significance threshold of ED ≥ 0.25, derived from the fitted values of all high-quality SNPs [34], was used to identify significant association peaks. The distribution of ED-associated values of SNPs on different chromosomes is displayed in Figure S1. This analysis defined five QTLs, which were designated as *qFERbin* QTLs, located on chromosome bins 1.06, 4.04, and 4.05 with a total length of 52.22 Mb, and 2507 genes. A total of 481 genes with non-synonymous SNP loci were identified from these five QTLs, and these QTLs were named *qFERbin*. For instance, *qFER1.06* denotes the QTL identified on chromosome bin 1.06. This QTL spanned a 5.98 Mb region on chromosome 1, ranging from 195,150,000 to 201,130,000 bp, and contained 458 genes. Simultaneously, two QTLs (*qFER4.04/4.05* and *qFER4.05*) were identified on chromosome 4 with intervals of less than 1.01 Mb (Table 1). The *qFER4.04/4.05* spanned an 18.16 Mb region (23,670,000–41,830,000 bp) and contained 1256 genes, while *qFER4.05* covered a 28.50 Mb region (42,840,000–71,340,000 bp) with 793 genes. Given the substantial genetic length and the magnitude of the ED peak on chromosome 4, we propose that this genomic region represents a hotspot for FER resistance in maize (Figure 3A).

### 2.3. BSA Mapping of GER Resistance

BSA was similarly conducted for GER resistance. Two DNA pools were constructed from F_2_ individuals exhibiting extreme GER phenotypes, derived from a cross between the resistant parent R227 and the susceptible parent S121. Whole-genome sequencing of the susceptible and resistant bulks generated 231,018,662 and 240,786,470 clean reads, corresponding to 69.20 Gb and 72.14 Gb of high-quality sequence (Q30 ≥ 93.19%), respectively (Table S1). Alignment of these reads to the reference genome resulted in mapping rates of 98.32% for the susceptible bulk and 98.14% for the resistant bulk (Table S1). A total of 10,476,096 SNPs were identified, and 3,152,820 high-quality SNPs were obtained, which were used for further association analysis (Table S2).

The association between allele frequency and GER resistance was evaluated by calculating the ED for each high-quality SNP between the GER_S and GER_R bulks. The ED values were plotted across all maize chromosomes (Figure 3B), with the full distribution provided in Figure S2. Using a significance threshold of ED ≥ 0.25, we identified 11 QTLs, which we designated as *qGERbin*, in chromosome bins 2.08–2.10, 4.05, and 4.06 (Table 2). These QTLs collectively spanned 73.94 Mb and encompassed 3261 genes, 764 of which contained non-synonymous SNPs.

The QTL *qGER2.09* spanned a 15.58 Mb region starting from 227,400,000 to 242,980,000 on chromosome 2, bin2.08–2.10, and contained 1429 genes (Table 2). Moreover, three QTLs were located on Chr 4, *qGER4.05-1*, *qGER4.05-2*, and *qGER4.06*, which spanned a region of 48.35 Mb, 6.12 Mb, and 6.34 Mb, starting from 58,580,000 to 106,930,000, 119,340,000 to 125,460,000, and 160,050,000 to 166,390,000, and contained 1063, 152, and 617 genes, respectively (Table 2). The intervals between adjacent QTLs on chromosome 4 were 12.41 Mb and 34.59 Mb, respectively. Given the high density and substantial genomic span of the QTLs on chromosome 4, we identified this chromosome as a major hotspot for GER resistance in maize (Figure 3B).

### 2.4. Candidate Genes and Enrichment Analysis

We further characterized each QTL and conducted KEGG pathway enrichment analysis for the identified genes residing in the QTL regions for FER and GER resistance, respectively. A total of 458, 1256, 793, 1429, 1063, 152, and 617 genes were identified in *qFER1.06*, *qFER4.04/4.05*, *qFER4.05*, *qGER2.09*, *qGER4.05-1*, *qGER4.05-2*, and *qGER4.06*, respectively (Table 1 and Table 2). KEGG pathways enriched in FER QTLs were identified as endocytosis, peroxisome, phagosome, phosphatidylinositol signaling, plant hormone signal transduction, ubiquitin-mediated proteolysis, plant–pathogen interaction, circadian rhythm–plant, biotin metabolism, citrate cycle, oxidative phosphorylation, carbohydrate metabolism, biosynthesis and metabolism of amino acids, fatty acid, ribosome, spliceosome, and the replication, splicing, repair, homologous recombination, transport and metabolism of nucleic acid (Figure S3). The similar enrichment results, which were without the circadian rhythm–plant, biotin metabolism, and citrate cycle but additionally enriched the propanoate metabolism and carotenoid biosynthesis, were exhibited in the GER QTLs (Figure S4).

**Figure 4:**
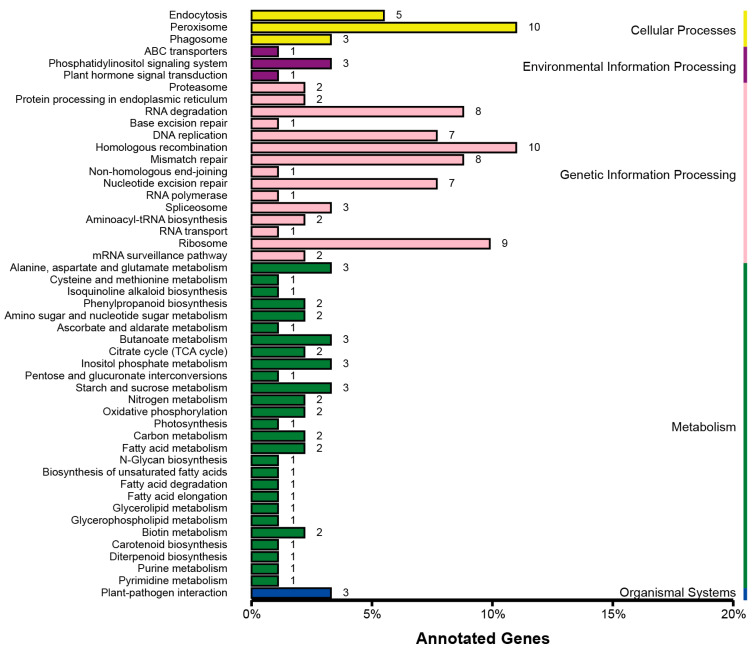
The KEGG_enrichment for the non-synonymous SNP-associated genes in FER QTL regions.

**Figure 5:**
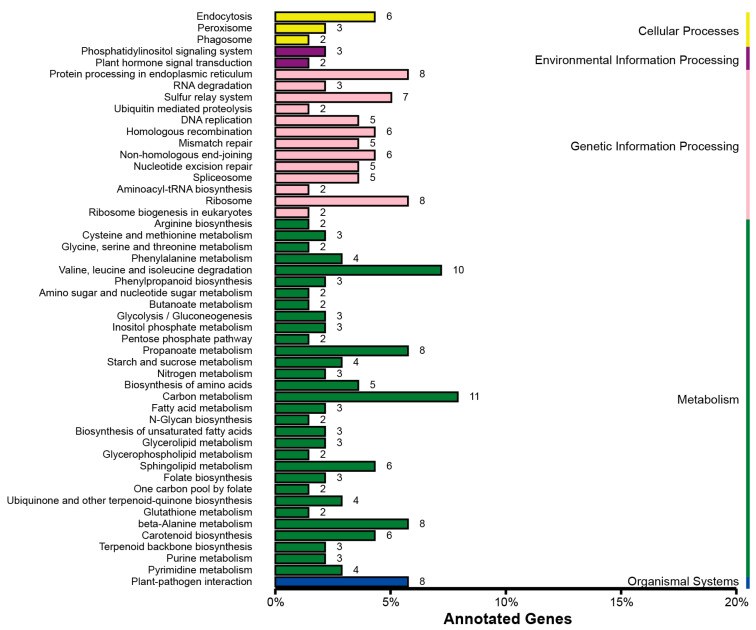
The KEGG_enrichment for the non-synonymous SNP-associated genes in GER QTL regions.

A total of 59,899 and 67,070 significant SNPs were identified from the extreme phenotype mixed pools for the FER and GER mapping populations, respectively. The identified SNPs were further screened for non-synonymous SNPs as candidate sites. A total of 350 and 391 non-synonymous SNPs were identified from the mixed pools for the two mapping populations. A total of 502 and 810 genes were closely associated with these SNPs. KEGG pathway enrichment analysis was further conducted for these non-synonymous SNP-associated genes. The non-synonymous SNP-associated genes in FER QTLs were enriched in endocytosis, peroxisome, phagosome, ABC transporters, phosphatidylinositol signaling, plant hormone signal transduction, ubiquitin-mediated proteolysis, plant–pathogen interaction, biotin metabolism, citrate cycle, oxidative phosphorylation, carbohydrate metabolism, biosynthesis and metabolism of amino acids and fatty acid, RNA degradation, ribosome, spliceosome, and DNA replication, mismatch repair, homologous recombination, nucleotide excision repair, and so on (Figure 4). Compared with the FER QTLs, more genes were enriched in the pathways of plant–pathogen interaction, folate and carotenoid biosynthesis, ubiquinone and other terpenoid–quinone biosyntheses, propanoate metabolism, sphingolipid metabolism, beta-alanine metabolism, and carbohydrate and amino acid metabolism for the GER QTLs (Figure 5).

A total of 52 genes were identified as cross genes that are associated with the non-synonymous SNPs in the overlap region of the QTLs for both FER and GER resistance. There were six genes without any annotation whose association with the ear rot resistance could not be determined, but 18 genes were possibly associated with the FER and GER resistance among the other 46 genes (Table 3). The annotation information regarding these genes suggested their involvement in various pathways, including enzyme activities such as protein serine/threonine kinase activity, transporter activity, transcription factor activity, electron transfer activity, cytoplasmic vesicle, integral component of organelles such as endoplasmic reticulum, ribosome, membrane, wall, thylakoid, and exocyst, proteolysis, protein phosphorylation, glycosylation, methylation, and ubiquitination, response to wounding and JA, defense response to bacterium, carotenoid and hydrogen peroxide biosynthesis, metal ion binding, heat shock protein binding, translation, exocytosis, oxidation-reduction, pollen development, DNA binding, and ATP binding pathway in this study.

### 2.5. Candidate Genes Expression Analysis

**Figure 6:**
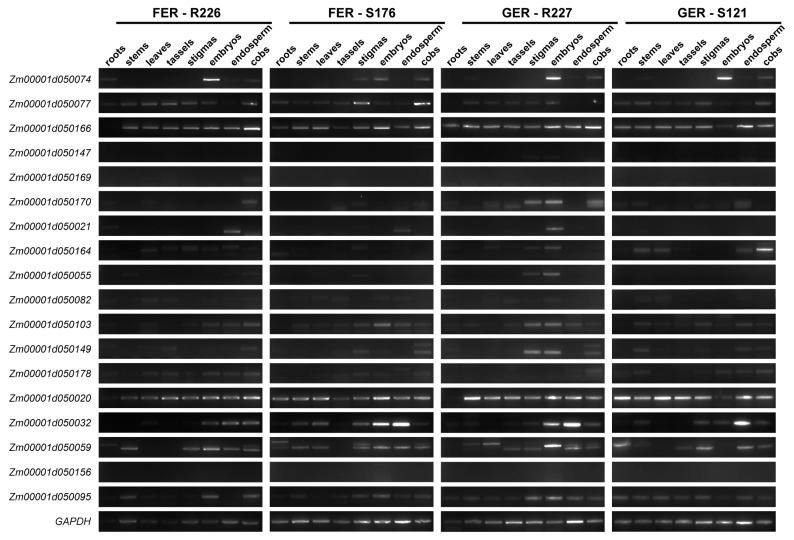
Tissue expression profiles of 18 candidate genes in various tissues at 15 days after pollination.

The tissue-specific expression profiles of 18 candidate genes (LRR-RLK, WAKL family members) in roots, stems, leaves, tassels, stigmas, embryos, endosperm, and cobs of maize (15 days after pollination) were detected by RT-PCR, with GAPDH as the internal reference gene. The results are as follows (Figure 6).

*Zm00001d050074* was mainly expressed in embryos, endosperm, and cobs, suggesting that it may be involved in the defense response of maize against ear rot pathogens. It showed relatively high expression levels in the embryos of maize lines R226, R227, and S121. *Zm00001d050077* and *Zm00001d050166* were expressed in all tested tissues. Specifically, *Zm00001d050077* had relatively higher expression in the stigmas and cobs of line S176, while *Zm00001d050166* showed slightly higher expression in the cobs of lines R226 and R227. *Zm00001d050170* was weakly expressed in the cobs of R226 and the stigmas of S176; it was also detected in the stigmas, embryos, and cobs of R227, with relatively higher expression in embryos. *Zm00001d050021* was only detected in the endosperm of the three maize lines (R226, S176, and R227). It exhibits tissue-specific expression and may be associated with resistance to ear rot. *Zm00001d050164* showed slightly higher expression in the endosperm of R227 and the cobs of S121, and no or weak expression in other lines and tissues. *Zm00001d050055* was weakly detected in the stems, endosperm, and cobs of R227, as well as the stigmas of S176; it had relatively higher expression in the stigmas and embryos of R227. *Zm00001d050082* showed no or weak expression in all tissues of all tested lines. *Zm00001d050103* was weakly detected in all tissues of all lines, with relatively higher expression in the embryos of S176 and the stigmas and embryos of R227. *Zm00001d050149* had higher expression in the stigmas and embryos of R227, and no or weak expression in other tissues. *Zm00001d050178* showed weak or no expression in all tissues of all tested lines. *Zm00001d050020* and *Zm00001d050095* were expressed in all tissues without significant tissue specificity or expression differences among different tissues and lines. *Zm00001d050032* was expressed in all tissues, with high expression levels in the endosperm of lines S176, R227, and S121. *Zm00001d050059* had relatively higher expression in the embryos of R227. In addition, no expression of *Zm00001d050147*, *Zm00001d050169*, and *Zm00001d050156* was detected in any of the tested tissues.

**Figure 7:**
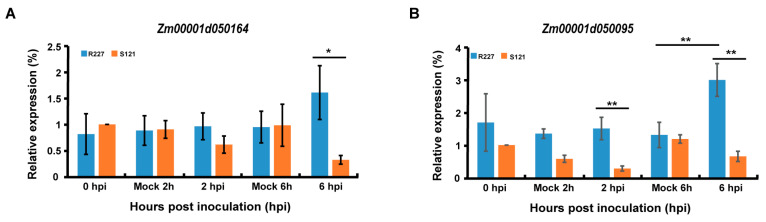
qRT-PCR results of candidate genes *Zm00001d050164* (**A**) and *Zm00001d050095* (**B**) in resistant and susceptible lines after Fusarium inoculation. * indicates significant difference at *p* < 0.05, ** indicates highly significant difference at *p* < 0.01.

Based on the results of tissue-specific expression analysis, eight candidate genes, including key genes such as *LRR-RLK* and *WAKL* family members, were selected for quantitative Real-Time PCR (qRT-PCR) detection to verify their expression dynamics under Fusarium stress. The results showed that the *Zm00001d050164* (a *WAKL* family gene) and *Zm00001d050095* had significant expression differences between resistant and susceptible lines at 6 hpi. Among them, *Zm00001d050095* was up-regulated in resistant lines after pathogen inoculation, which is closely related to the immune response of maize, further supporting the potential role of these two genes in resisting ear rot (Figure 7A,B). For the other candidate genes, no significant expression differences were detected between the inoculated group and the control group, or between resistant and susceptible lines.

## 3. Discussion

Ear rot has emerged as a major threat to maize production in China. Developing cultivars with enhanced resistance to FER and GER is becoming a critical need. The mining of stable and simultaneously resistant genes/loci to FER and GER will significantly accelerate breeding programs.

Compared with the classical linkage mapping with RIL/DH populations, BSA provides faster results (weeks vs. years for RIL development) and requires fewer resources. Meanwhile, BSA also has its limitations: the QTL intervals identified by the BSA are relatively broad. This limitation is inherent to BSA methodology, which provides rapid identification of genomic regions but with lower resolution, mainly due to the limited number of recombination events captured in the bulks and the lack of individual genotypic data. These broad intervals should be considered as primary candidate regions for fine-mapping validation. BSA based on pooled sequencing generally yields relatively wide QTL confidence intervals,

Many efforts have been made to identify QTLs associated with FER and GER resistance. The locus on chromosome bin 1.06 resistant to FER has been validated by two linkage mapping studies using an F_2_ population and a RIL population, respectively [35,36]. The QTL intervals were 95.03 Mb (110.78–205.81 Mb) and 2 Mb (192–194 Mb), with corresponding PVEs of 5.45 and 5.76 (Table S3). Similarly, the QTL *qFER4.04-4.05* has been reported in multiple studies, including two inbred line populations and one F_2_ population [25,35,37]. The GWAS results from the two natural populations identified one and three associated markers at 29.04 Mb and 29.96 Mb in bin4.04, with PVEs of 1.60, 3.65, 4.83, and 3.64 (Table S3). And the QTL detected in the F_2_ population spanned 35.53 Mb (32.89–68.42 Mb), covering part of both *qFER4.04–4.05* and *qFER4.05*, which were identified in the present study, with a PVE of 4.46 (Table S3). In addition to being covered by the above-mentioned QTL, *qFER4.05* has also been reported in at least one F_2_ population (PVE = 17.95) and one RIL population (PVE = 2.78) (Table S3) [14,35,38]. These QTLs were repeatedly detected across different environments in independent studies, indicating that they are relatively stable QTLs. For GER resistance, the locus on chromosome bin 2.09 has been reported by two linkage mapping studies and one association mapping study using one RIL population and two DH populations [10,31,39]. The intervals from linkage mapping were 12.59 Mb (220.44–233.03 Mb, B73_V3) and 4.19 Mb (225.87–230.06 Mb), which partially overlapped with *qGER2.09* in this study, with corresponding PVEs of 29.40 and 6.90 (Table S3). The marker identified by GWAS was located at 238.01 Mb, with a PVE of 2.84 (Table S3). Another linkage mapping study using an RIL population detected a QTL on bin 4.02–4.05 spanning 129.66 Mb (5.87–135.53 Mb), which completely covered the two QTLs *qGER4.05-1* and *qGER4.05-2* identified in this study, with a PVE of 11.00 (Table S3) [10]. In contrast, a separate linkage mapping study using an RIL population detected a QTL on bin 4.05 spanning 51.52 Mb (24.28–75.54 Mb), which shared a 16.96 Mb overlapping region (58.58–75.54 Mb) with *qGER4.05-1* in the present study, with a PVE of 11.00 (Table S3) [29]. And this QTL was detected in three distinct environments, suggesting that it is a stable QTL. Surprisingly, the QTL *qGER4.06* detected in this study has not been reported in previous studies, suggesting new resistant loci for GER resistance. A promising strategy for enhancing the durability of ear rot resistance is the pyramiding of multiple resistance genes into elite inbred lines to develop multi-resistant cultivars. In this context, the novel QTLs identified here represent promising candidates for future breeding programs aimed at achieving broad-spectrum resistance. Their integration, particularly alongside the stable, co-localized FER/GER QTLs on chromosome 4, could provide valuable donor resources and contribute to the development of more resilient maize varieties.

On the other hand, a few studies simultaneously conducted phenotype identification and QTL mapping for FER and GER resistance at the same location. Proverbially, infections caused by pathogens of maize ear rot are complex and diverse because of their diverse pathogenic infection pathways and the sensitivity to the environmental conditions [35]. This study simultaneously investigated the underlying QTL resistances to both FER and GER under shared environmental conditions. By cultivating two distinct mapping populations at the same location and time and employing artificial inoculation, we ensured that both populations experienced nearly identical post-inoculation conditions. This design enhances the reliability of directly comparing the genetic architectures of FER and GER resistance. Under these standardized conditions, three QTLs for FER and four for GER were detected. Notably, a QTL on bin 4.05 was identified for both diseases. This genomic region has been frequently associated with resistance in previous studies targeting either FER or GER [10,14,25,29,35,37,38], suggesting that resistant loci on bin 4.05 might be resistance hotspots for both FER and GER. Furthermore, the specific QTLs *qFER4.05* and *qGER4.05-1* were found to overlap within a 58.58–71.34 Mb interval on bin 4.05. This co-localization strongly indicates the presence of a gene or cluster of genes within this region that confers resistance to both pathogens. Such a locus would be highly valuable for maize breeding programs aimed at pyramiding multiple disease resistance genes. Therefore, we selected this overlapping region for fine mapping. Subsequent genetic analysis identified 18 candidate genes within this interval that are potentially involved in the defense responses against FER and GER, providing a focused set of targets for subsequent functional validation.

Plant immune response was activated by the perception of pathogen-associated molecular patterns (PAMPs) or effector proteins, and this process was mediated by pattern-recognition receptors (PRRs) and nucleotide-binding and leucine-rich repeat domain-containing receptors (NLRs). Plant R-genes, which include the leucine-rich repeat (LRR) and leucine-rich repeat receptor-like kinase (LRR-RLK) gene families, the main type of proteins encoded by which are NLRs, are known to regulate plant resistance against various biotic stresses. Two *LRR-RLKs* (*Zm00001d050074* and *Zm00001d050077*) and one *LRR* gene (*Zm00001d050166*) were located in this region, which have been reported to regulate disease resistance in gramineae plants [40,41,42,43]. Notably, a recent study indicated that the *LRR-RLK* genes were potentially involved in resistance to *F. verticillioides* infection in maize [44].

*Zm00001d050147* encodes a remorin protein in this region. Two maize remorins, *ZmREM6.3* and *ZmREM1.3*, have been demonstrated to exhibit resistance against Northern Corn Leaf Blight (NCLB) and Southern Corn Rust (SCR), respectively, likely via SA/JA-mediated defense signaling pathways [45,46]. The presence of a remorin in our candidate interval suggests a possible conserved role for this protein family in broad-spectrum disease resistance in maize.

Furthermore, *Zm00001d050169* and *Zm00001d050170* were annotated as cytochrome P450 monooxygenases. This class of enzymes is directly relevant to *F. graminearum* defense; a wheat cytochrome P450 was shown to enhance resistance to deoxynivalenol (DON), a key mycotoxin and virulence factor produced by this pathogen during Fusarium Head Blight [47]. Given that *F. graminearum* is also the causal agent of GER in maize, these cytochrome P450s are compelling candidates for mediating resistance to the fungus or its toxins. This group also includes a gene, *Zm00001d050021*, for abscisic acid 8′-hydroxylase, which is itself a cytochrome P450 monooxygenase [48,49], linking a key hormone regulatory pathway to the cytochrome P450 activity in this locus.

The wall-associated receptor kinases (WAKs) and WAK-likes (WAKLs) have been reported to be associated with resistance to plant diseases, such as NCLB, maize Gibberella Stalk Rot (GSR) and Head Smut (HS), wheat rust, Fusarium Crown Rot (FCR) and Sharp Eyespot, Barley Stripe (BS), and Rice Blast (RB) [50,51,52,53,54,55,56]. The candidate gene *Zm00001d050164* in this region encoded a precursor of WAKL20, which might be related to resistance to maize ear rot [55,56]. Moreover, four genes (*Zm00001d050055*, *Zm00001d050082*, *Zm00001d050103*, and *Zm00001d050149*) in this region encoded glycine-rich cell-wall structural protein-like, pectinesterase, trichome birefringence-like protein, and precursor of exopolygalacturonase, respectively, and might regulate the defense against pathogens by altering the structure of the cell wall or interfering with the hypersensitive defense response [57,58,59,60,61]. Another candidate gene, *Zm00001d050178*, encoded a protein that was homologous to the N-terminal of Xylanase inhibitors, which have been reported to be associated with resistance to wheat FHB [62]. *Zm00001d050020*, *Zm00001d050032*, and *Zm00001d050059* encoded proteins that are related to post-translational glycosylation and methylation modifications, and these post-translational modifications have been reported to regulate disease resistance in maize, rice, and wheat [63,64,65]. *Zm00001d050156* encoded a pentatricopeptide repeat-containing protein (PPR), while the PPRs have been reported to regulate plant responses to abiotic stresses in rice [66]. And the *Zm00001d050095* encoded Exo70 exocyst complex subunit, which has been reported to participate in rice immunity [67].

The tissue-specific expression analysis and the qRT-PCR results of candidate genes (including *LRR-RLK* and *WAKL* family members) under Fusarium stress supplemented the functional exploration of maize ear rot resistance-related genes. Among them, *Zm00001d050164* (a *WAKL* gene) and *Zm00001d050095* showed significant expression differences between resistant and susceptible lines at 6 hpi. *WAKL* genes are key in plant biotic stress responses by mediating cell-wall signal transduction, so *Zm00001d050164* may participate in the early recognition of ear rot pathogens. *Zm00001d050095* was up-regulated in resistant inbred lines after inoculation, indicating it may be closely related to maize resistance against ear rot. Consistent with tissue-specific expression results, *Zm00001d050164* was slightly more expressed in R227′s endosperm and S121′s cobs, while *Zm00001d050095* had no obvious tissue specificity. Their expression differences under stress are closely related to material resistance, suggesting they may play a direct role in whole-plant resistance responses to ear rot.

Notably, the other candidate genes showed no significant expression differences between the inoculated and control groups, or between resistant and susceptible lines. This may be due to three reasons: these genes are not involved in the early response to ear rot pathogens; their expression is regulated by unmet environmental or physiological conditions; or their function depends on post-transcriptional modification rather than transcriptional changes, which cannot be detected by qRT-PCR.

Future research should focus on the functional validation of the candidate genes within the identified QTLs, especially *Zm00001d050164* and *Zm00001d050095*, which will be crucial to move from genetic association to a mechanistic understanding of ear rot resistance. A comprehensive understanding of the functions of these genes will not only illuminate the complex mechanisms of quantitative disease resistance in maize but will also directly empower marker-assisted breeding strategies. The translation of this knowledge into elite germplasm is a vital step toward developing durable resistance against devastating ear rot diseases.

## 4. Materials and Methods

### 4.1. Plant Materials and Field Management

In the previous research, we identified two highly resistant inbred lines, R226 and R227, with resistance to FER and GER, respectively. These two inbred lines were used as the resistant parent in this study. Two highly susceptible inbred lines, S176 and S121, were selected to be the susceptible parents. Using R226 and S176, R227 and S121, two F_2_ populations (including 168 and 185 individuals, respectively) and their corresponding F_2:3_ families were constructed for QTL mapping. The parental lines, F_1_ hybrid lines, and F_2_ individuals were planted at two field trial locations, Chengdu and Xichang, in Sichuan Province, China. The corresponding F_2:3_ families, which were selected from highly resistant F_2_ individuals, were planted at Yingzhou, in Hainan Province, China. Each line was grown in single rows of 5 m, with 0.25 m spaces between plants within a row and a planting density of 67,000 plants/ha, and standard cultural practices were followed throughout the growth period. All plants were artificially bag-pollinated and inoculated with a pathogen spore suspension.

### 4.2. Pathogen Culture and Artificial Inoculation

The *F. verticillioides* and *F. graminearum* strains were isolated and stored by our laboratory, and they were used for artificial inoculation. Firstly, the pathogens were inoculated on a potato dextrose agar medium in plates and cultured in a dark incubator for 4–5 days at 25 °C until the hyphae filled the medium plates, and they were stored in the refrigerator at 4 °C for future use. Then, each plate with the pathogen was cut into fungal discs with a diameter of 0.5 cm, and 3 discs were suspended in 100 mL potato dextrose liquid medium before being shaken at 180 rpm for 10–15 days at 25 °C; then, the pathogen liquid was gathered and stored in the refrigerator at 4 °C for future use. Before artificial inoculation, the spores were collected after the hyphae were filtered, and a spore suspension with a concentration of 5 × 10^6^ spores/mL was prepared with sterile distilled water; tween-80 (Solarbio, Beijing, China) (2%) was added to the spore suspension and mixed, and the spores were ready for inoculation. The silk channel injection method, which more closely resembles natural infection of a non-wounded host plant, was used for artificial inoculation, and the best differentiation between resistant and susceptible genotypes was easier to obtain when inoculation occurred within a week after silking [2,68,69]. In this study, two days after artificial pollination, 2 mL of the prepared pathogen spore suspension was injected into the silk channel in two consecutive injections with a syringe. After inoculation, the wound was pinched gently with fingers to prevent the pathogen spore suspension from flowing out. At the maturation stage, maize ears were manually removed from the husks and harvested, ear rot incidence severity was surveyed, and resistance scales were evaluated.

### 4.3. Disease Evaluation

Individual plants were artificially inoculated with *F. verticillioides* and *F. graminearum*, and evaluated for their resistance to FER and GER at different field trial locations. An evaluation system with five grades (1, 3, 5, 7, and 9) was used to visually evaluate the severity of ear rot disease. Based on the size of the lesion area due to ear rot damage, grades 1, 3, 5, 7, and 9 represented 0 to 1%, 2 to 10%, 11 to 25%, 26 to 50%, and 51 to 100%, respectively, of damage. Each individual plant was independently rated at the maturation stage.

### 4.4. DNA Extraction, Library Construction, and Sequencing

Allele frequency estimates in BSA are dependent on the variation in segregant samples and sequencing technology. The variation due to segregant samples can be minimized by either increasing the number of segregants or the bulk size [70]. According to the phenotype of each population in multiple environments, the parental lines R226 and S176 and their F_2_ population, 22 resistant and 22 susceptible F_2_ individuals, which were resistant to FER and planted at Xichang, were selected to construct BSA pools. The parental lines R227 and S121 and their F_2_ populations, 27 resistant and 27 susceptible F_2_ individuals, which were resistant to GER and planted at Xichang, were selected to construct BSA pools. Genomic DNA was extracted individually by the high-throughput cetyltrimethylammonium bromide (CTAB) method, and an equal amount of the extraction from each individual was mixed to form resistant and susceptible DNA pools; the DNA extracted from parental lines was also prepared for library construction. All of these DNA samples were sent to Biomarker Technologies (Beijing, China) for re-sequencing. The DNA samples were randomly broken into 350 bp fragments using ultrasonic fragmentation, and each fragment was repaired at the end, and A was added at the 3′ end; sequencing adapters are added, purified, and PCR-amplified to complete the construction of the sequencing library. Libraries built for each pool were sequenced using Illumina HiSeq after passing the quality inspection. The raw sequencing data (whole-genome sequencing for bulked segregant analysis of FER and GER traits in Zea mays) have been deposited in the NCBI Sequence Read Archive (SRA) under the BioProject accession PRJNA1417795 (http://www.ncbi.nlm.nih.gov/bioproject/1417795, accessed on 2 February 2026), with the corresponding BioSample accessions SAMN55017278–SAMN55017285 and SRA accessions SRR37076531–SRR37076538. The data will be released to the public on 1 March 2026.

### 4.5. Data Processing and Analysis

For data analysis, we filtered out the low-quality reads, which included multiple alleles, fewer than 4 supporting reads, those consistent with a mixed pool, and susceptible pool loci that were not from the susceptible parent, and then we aligned the clean reads to the reference maize genome (Maize B73 RefGen_v4) by using the bwa software (version 0.7.17) [71]. Picard tools were used to exclude the mark duplicates, and the GATK was used for preprocessing, including local realignment and base recalibration to ensure the accuracy of detected SNPs [8]. Then, GATK was used for variant (SNP and InDel) calling, filtering, and obtaining the final set of variant loci [8]. The parents were used to filter polymorphic information.

The Euclidean distance (ED) algorithm, which was used to predict the ED between two vectors defined by the frequencies of the alternate and the reference alleles in the high and low bulks to identify the region of interest [34,72], was used to estimate the difference between the two mixed DNA pools using the following formula:

$$\mathrm{ED}=\sqrt{{(A_{\mathrm{mut}}\;-A_{\mathrm{wt}})}^{2}\;+\;(C_{\mathrm{mut}}\;-{C_{\mathrm{wt}})}^{2}\;+(G_{\mathrm{mut}}\;-{\;G_{\mathrm{wt}})}^{2}+\;(T_{\mathrm{mut}}\;-{\;T_{\mathrm{wt}})}^{2}}$$

where *A_mut_*, *C_mut_*, *G_mut_*, and *T_mut_* were used to represent frequencies of A, C, G, and T bases in the resistant pool, while *A_wt_*, *C_wt_*, *G_wt_*, and *T_wt_* were used to represent the frequencies in the susceptible pool, respectively. The ED value represents the size of the difference between the two DNA pools. Higher ED values indicate larger allelic frequency differences between the two extreme bulks and thus stronger genetic effects of the corresponding QTL.

The significance threshold of ED greater than or equal to 0.25 was derived from the fitted values of all high-quality SNPs following established methods in BSA [34]. This threshold represents the 95th percentile of the ED distribution under the null hypothesis of no association [34].

### 4.6. Pathway Enrichment Analysis

Genes usually interact with each other to play roles in certain biological functions. Pathway-based analysis usually helps to further understand genes’ biological functions, and the pathway enrichment analysis identified significantly enriched metabolic pathways or signal transduction pathways in genes in defined QTLs. KEGG, which was the major public pathway-related database [73], was used to perform the pathway enrichment analysis for genes in the identified QTLs according to the *p*-value.

### 4.7. Tissue-Specific Expression Analysis

To clarify the spatial expression characteristics of candidate genes in maize, we performed tissue-specific expression analysis using RT-PCR. The two resistant lines (R226 and R227) and susceptible lines (S176 and S121) were selected, key tissues (roots, stems, leaves, tassels, stigmas, embryos, endosperm, and cobs) at 15 days after pollination were collected from healthy plants (3 biological replicates each), immediately frozen in liquid nitrogen, and stored at −80 °C. Total RNA was extracted using Trizol reagent (Invitrogen, Carlsbad, CA, USA), and its integrity and purity were verified; cDNA was synthesized via reverse transcription. The concentration of PCR products of the internal reference gene GAPDH was used for normalization to ensure equal loading of cDNA templates, followed by RT-PCR amplification of candidate genes. Primers used for RT-PCR and qRT-PCR were provided in Supplementary Materials Table S4.

### 4.8. qRT-PCR Analysis

To verify the expression dynamics of candidate genes under pathogen stress, qRT-PCR analysis was performed. The resistant lines R227 and susceptible lines S121 were selected, maize ears were inoculated with *F. graminearum* (1 × 10^6^ conidia/mL) and sterile water (blank control) at 15 days after pollination. Ear samples were collected at 0 h (CK), 2 h, and 6 h post-inoculation and stored at −80 °C. Total RNA extraction and cDNA synthesis followed the same protocol as above; specific primers were designed and verified, and qRT-PCR was conducted using Taq ProUniversal SYBR qPCR Master Mix (Vazyme, Nanjing, China). The 2^−ΔΔCt^ method was used to calculate relative expression levels, with TTEST for significance analysis (*p* < 0.05).

## 5. Conclusions

In conclusion, we employed BSA on two F_2_ mapping populations to identify QTLs conferring resistance to FER and GER and identified five and eleven QTLs for FER and GER, respectively. We found that chromosome 4 was identified as a major hotspot for resistance to both diseases, and the co-localization of the FER QTL (*qFER4.05*) and GER QTL (*qGER4.05-1*) within a 58.58–71.34 Mb interval on bin 4.05, suggesting a potential locus for broad-spectrum resistance. Within this overlapping region, we identified 18 high-confidence candidate genes, including several genes that have established roles in plant defense. The tissue-specific expression analysis and the qRT-PCR results of candidate genes showed that the *Zm00001d050164* (a *WAKL* family gene) and *Zm00001d050095* had significant expression differences between resistant and susceptible lines, and *Zm00001d050095* was up-regulated in resistant lines after pathogen inoculation, which is closely related to the immune response of maize, further supporting the potential role of these two genes in resisting ear rot. Our results reveal novel resistance QTLs, provide a valuable set of candidate genes for functional validation, and advance the understanding of the genetic architecture of ear rot resistance, providing critical resources for marker-assisted breeding to develop maize hybrids with durable resistance to both FER and GER.

## Figures and Tables

**Table 1:** Statistics for QTLs identified for FER resistance.

QTLs	Bins	Chr.	Start (Mb)	End (Mb)	Size (Mb)	Gene_NO.	Peak ED
*qFER1.06*	1.06	1	195.15	201.13	5.98	458	0.26
*qFER4.04*	4.04–4.05	4	23.67	41.83	18.16	1256	0.34
*qFER4.05*	4.05	4	42.84	71.34	28.50	793	0.28

Chr. = chromosome, and Gene_NO. = Gene_Number.

**Table 2:** Statistics for QTLs identified for GER resistance.

QTLs	Bins	Chr.	Start (Mb)	End (Mb)	Size (Mb)	Gene_NO.	Peak ED
*qGER2.09*	2.08–2.10	2	227.40	242.98	15.58	1429	0.20
*qGER4.05-1*	4.05	4	58.58	106.93	48.35	1063	0.18
*qGER4.05-2*	4.05	4	119.34	125.46	6.12	152	0.17
*qGER4.06*	4.06	4	160.05	166.39	6.34	617	0.18

Chr. = chromosome, and Gene_NO. = Gene_Number.

**Table 3:** Distribution of candidate genes associated with resistance to FER and GER in the co-localization region on chromosome 4.

Gene_ID	Start (bp)	End (bp)	Symbol	Description	Annotated Databases
*Zm00001d050074*	64,147,004	64,152,219	LRR-RLK	Leucine-rich repeats receptor-like protein kinase (Precursor)	Pfam/Swissprot
*Zm00001d050077*	64,429,793	64,431,264	LRR-RLK	Leucine-rich repeats receptor-like protein kinase (Precursor)	Pfam/Swissprot
*Zm00001d050166*	70,207,743	70,219,453	SKIP1	F-box protein SKIP1	Swissprot
*Zm00001d050147*	69,181,573	69,184,287	/	Remorin, C-terminal region	Pfam
*Zm00001d050169*	70,238,029	70,244,611	CYP86B1	Cytochrome P450 86B1	Swissprot
*Zm00001d050170*	70,328,124	70,329,750	CYP86B1L	Cytochrome P450 86B1-like	nr
*Zm00001d050021*	60,281,924	60,287,213	ABA8′-H	Abscisic acid 8′-hydroxylase	KEGG
*Zm00001d050164*	70,178,212	70,181,943	WAKL20	Wall-associated receptor kinase-like 20 (precursor)	Swissprot
*Zm00001d050055*	62,771,016	62,771,591	GRPL	glycine-rich cell wall structural protein-like	nr
*Zm00001d050082*	64,847,021	64,848,362	PPE8B	Pectinesterase/pectinesterase PPE8B	KEGG
*Zm00001d050103*	65,733,096	65,736,167	TBL16	Protein trichome birefringence-like 16	Swissprot
*Zm00001d050149*	69,353,358	69,355,399	/	Exopolygalacturonase (precursor; fragment)	Swissprot
*Zm00001d050178*	71,005,585	71,008,083	ASPG2	Xylanase inhibitor N-terminal	Pfam
*Zm00001d050020*	60,146,877	60,161,157	FUT11	Glycoprotein 3-alpha-L-fucosyltransferase	KEGG
*Zm00001d050032*	61,315,575	61,321,274	G1PAT	Glucose-1-phosphate adenylyltransferase	KEGG
*Zm00001d050059*	62,917,785	62,921,893	ICMT	Protein-S-isoprenylcysteine O-methyltransferase	KEGG
*Zm00001d050156*	69,627,916	69,629,499	PCMP-H12	Pentatricopeptide repeat-containing protein At1g08070	Swissprot
*Zm00001d050095*	65,387,724	65,389,604	EXO70B1	Exo70 exocyst complex subunit	Pfam

## Data Availability

All relevant data are within the manuscript and its Supplementary Files.

## Supplementary Materials

The following supporting information can be downloaded at: https://www.mdpi.com/article/10.3390/plants15091401/s1. Figure S1. The distribution of ED_values for high-quality SNPs to the FER resistance on different chromosomes. The ED value represents the size of the difference between the FER_S and FER_R DAN pools. The red dashed line represents the correlation threshold, which was calculated as 0.25. Figure S2. The distribution of ED_values for high-quality SNPs to the GER resistance on different chromosomes. The ED value represents the size of the difference between the GER_S and GER_R DAN pools. The red dashed line represents the correlation threshold, which was calculated as 0.25. Figure S3. The KEGG_enrichment for whole genes in FER QTL regions. Figure S4. The KEGG_enrichment for whole genes in GER QTL regions. Table S1. Quality of whole-genome sequencing for the susceptible and resistant bulks and their parental lines. Table S2. Filter the high-quality loci for the sequencing data. Table S3. Synthetic Table of QTL Overlap with Previously Reported Maize Ear Rot Resistance QTLs. Table S4. Primers used for RT-PCR and qRT-PCR detection of candidate genes.
