# Supplementary material for: Bulked Segregant Analysis Revealed the Common Resistant QTLs Associated with Fusarium Ear Rot and Gibberella Ear Rot in Maize
Source: Plants (Basel). 2026 May 4;15(9):1401. doi: 10.3390/plants15091401 (PMC13164825; doi:10.3390/plants15091401)
Supplement: Supplementary file 1 [file plants-15-01401-s001.zip › plants-4163403-supplementary.pdf]

Supplementary materials

Table S1 Quality of the whole-genome sequencing for the susceptible and resistant bulks and their parental lines.

Populations	ID	Raw_Read	Clean_Reads	Clean_Base	Q30(%)	GC(%)	Total_reads	Mapped(%)	Properly_mapped(%)
Population_FER	FER_S	88,717,362	88,576,956	26,532,617,054	92.87	47.51	177,153,912	98.57	87.5
	FER_R	88,937,563	88,803,981	26,602,803,222	93.55	47.46	177,607,962	94.01	84.37
	FER_S pool	185,566,816	185,305,948	55,513,993,090	93.42	47.35	370,611,896	97.42	87.84
	FER_R pool	188,094,258	187,834,729	56,271,194,984	93.44	47.4	375,669,458	98.58	88.74
Population_GER	GER_S	84,538,047	84,403,460	25,283,466,758	93.2	46.98	168,806,920	98.39	88.64
	GER_R	88,937,980	88,835,605	26,614,809,896	93.38	47.39	177,671,210	98.69	89.07
	GER_S pool	231,381,728	231,018,662	69,204,052,706	93.19	47.28	462,037,324	98.32	88.28
	GER_R pool	241,143,830	240,786,470	72,135,105,008	93.30	47.48	481,572,940	98.14	88.39

ID = sequencing sample ID; Raw_Read = raw sequencing reads count; Clean_Reads = remaining reads count after filtering; Q30(%) = percentage of bases with a quality score ≥ 30 relative to the total number of bases; GC(%) = GC content percentage; Mapped(%) = mapping rate of the reference genome; Properly_mapped = paired-end reads that are are both mapped to the reference genome with insert sizes consistent with the sequencing fragment length distribution.

Table S2 Filter the high-quality loci for the sequencing data.

Populations	Total_SNPs	Multiple allele _SNPs	Reads support < 4_SNPs	Consistent with mixed pool_SNPs	Using parental filtered_SNPs	High quality_SNPs
Population _ FER	10,714,248	27,225	2,802,106	1,945,687	3,081,908	2,857,322
Population _ GER	10,476,096	26,974	1,674,901	2,472,833	3,148,568	3,152,820

Table S3 Synthetic Table of QTL Overlap with Previously Reported Maize Ear Rot Resistance QTLs

QTLs	Bins	Intervals (Mb)	Size(Mb)	Peak ED	Referense	Population	Type of QTL Mapping	QTL/SNP bins	Intervals	Size(Mb)	PVE
qFER1.06	1.06	195.15-201.13	5.98	0.26	Wen et al. 2021 [35]	F_2 population	Linkage mapping	bin1.05-1.06	110.78-205.81 Mb (B73_V4)	95.03	5.45
					Xia et al. 2022 [36]	RIL population	Linkage mapping	bin1.06	192.00-194.00 Mb (B73_V3)	2	5.76
qFER4.04	4.04-4.05	23.67-41.83	18.16	0.34	Chen et al. 2016 [25]	inbred lines	GWAS	bin4.04	29.035 Mb (B73_V1)	/	1.60
					Wen et al. 2021 [35]	F_2 population	Linkage mapping	bin4.03-4.05	32.89-68.42 Mb (B73_V4)	35.53	4.46
					Guo et al. 2020 [37]	Inbred lines	GWAS	bin4.04	29.96 Mb (B73_V3)	/	3.65/4.83/3.64
qFER4.05	4.05	42.84-71.34	28.50	0.28	Chen et al. 2012 [14]	$F_{2:3}$ population	Linkage mapping	bin4.05-4.06	/	/	17.95
					Wen et al. 2021 [35]	F_2 population	Linkage mapping	bin4.03-4.05	32.89-68.42 Mb (B73_V4)	35.53	4.46
					Wu et al. 2020 [38]	RIL population	Linkage mapping	bin4.05-4.06	/	/	2.78
qGER2.09	2.08-2.10	227.40-242.98	15.58	0.20	Ali et al. 2005 [10]	RIL population	Linkage mapping	bin2.08-2.09	220.44-233.03 Mb (B73_V3)	12.59	29.40
					Gaikpa, D.S. et al. 2021 [31]	DH population	GWAS	bin2.09	238.01 Mb (B73_V4)	/	2.84
					Martin, M. et al. 2012 [39]	DH population	Linkage mapping	bin2.09	225.87-230.06 Mb (B73_V4)	4.19	6.90
qGER4.05-1	4.05	58.58-106.93	48.35	0.18	Ali et al. 2005 [10]	RIL population	Linkage mapping	bin4.02-4.05	5.87-135.53Mb (B73_V4)	129.66	11.00
					Zhou et al. 2021 [29]	RIL population	Linkage mapping	bin4.05	24.28-75.54 Mb (B73_V4)	51.52	2.05/5.92/5.24
qGER4.05-2	4.05	119.34-125.46	6.12	0.17	Ali et al. 2005 [10]	RIL population	Linkage mapping	bin4.02-4.05	5.87-135.53Mb (B73_V4)	129.66	11.00
qGER4.06	4.06	160.05-166.39	6.34	0.18	/	/	/	/	/	/	/

Table S4 Primers used for RT-PCR and qRT-PCR detection of candidate genes

Gene_ID	Primers-F	Primers-R
Zm00001d050074	ATAGATTCAGCGGAGGTTTGC	AGCGTAGACTTCGCAATGTAGC
Zm00001d050077	GCCCCTTGGTCCAGATAGATT	AACTTGGGCAGTCCAGAACAC
Zm00001d050166	GGCAACAGGGAAGCCATTA	CTTCACCAGCGTCACCAGA
Zm00001d050147	TTGTTTGACGACGCTGAGATT	GCCATTGACCTTGCTCCTCT
Zm00001d050169	GCAAGGACAGCCATGAGTTTAG	TCCAGAAGCGGCAGGTAGAG
Zm00001d050170	CCTTCTTCCGTCAAATCGTC	GCAAAGTTCCTCTTCTCCACC
Zm00001d050021	CATCGTCTCCCTCACTCTGC	GTCTTGAACACCTCGCCGTA
Zm00001d050164	CGCACCGCCAGGAACTT	GCACTTGACGGCCACCAC
Zm00001d050055	ACATTGCCCGAAAAGACCTG	GCCGATGAGCCTGGAGC
Zm00001d050082	CGTGAGGAAGGGCGTGTA	TGGCGCTGCGATAGGTG
Zm00001d050103	AGACCGAGCGACCAGTTACG	ATGACGGCGGTGTTCCTG
Zm00001d050149	GGTTCGGCAACGACTGGA	CTCGTGGGCAGCACTTTG
Zm00001d050178	TCCCTCACTGCCATCCCT	ACGCTGATGCCCTGTCCT
Zm00001d050020	ACTCTGGGAGCCTTGGAATC	GTTGCGTTAGACCGACTGG
Zm00001d050032	GTGGTTGTTTGAGGAGCATAAT	CATCGGTAGGGCAGCAAC
Zm00001d050059	CATTGCTGGAACGCCTGA	AAAGAATCGCCACAAGACCAA
Zm00001d050156	CCTCCGGGCGCTAATCA	CCGTCCAGGAAACCACGT
Zm00001d050095	TGCCAGCATACCAGTCATTCC	CCCTCAAAGAATCACCCACA

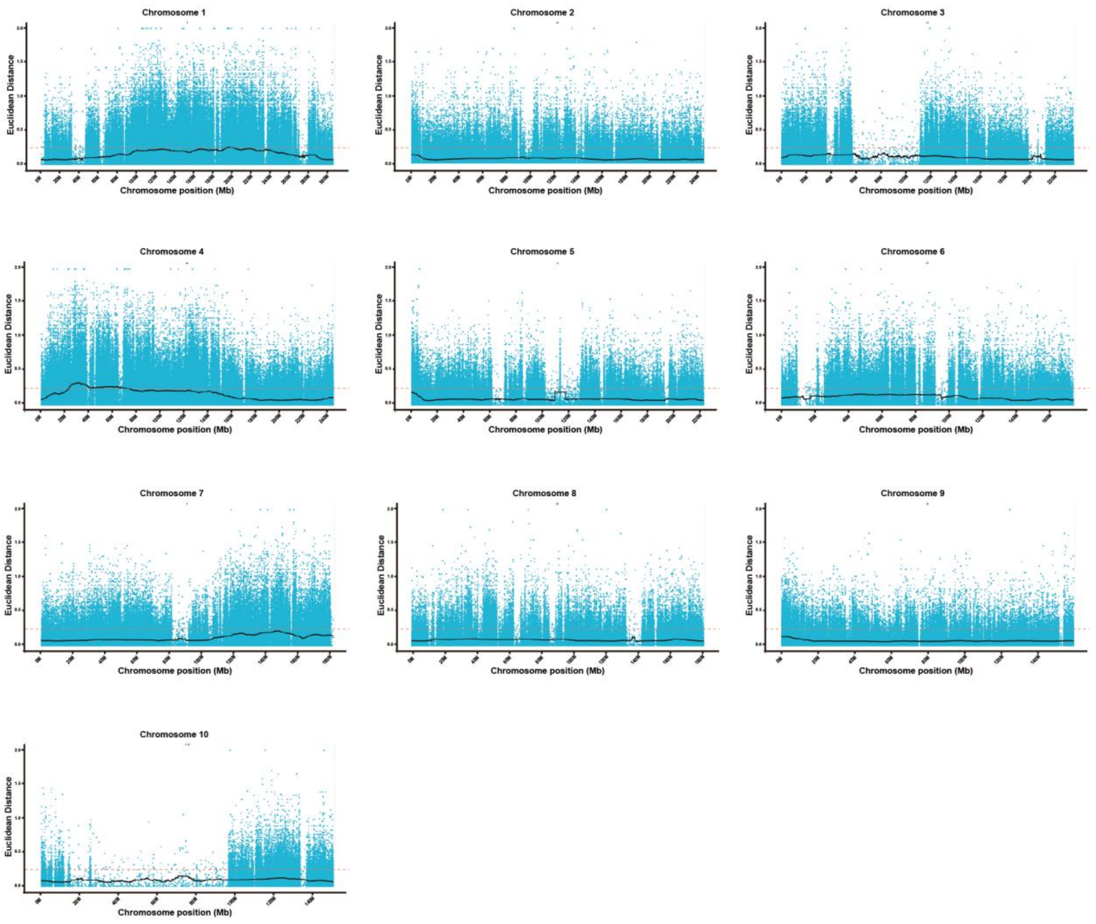

Figure S1 The distribution of ED_values for high-quality SNPs to the FER resistance on different chromosome. The ED value represents the size of the difference between the FER_S and FER_R DAN pools. The red dashed line represent the correlation threshold was calculated as 0.25.

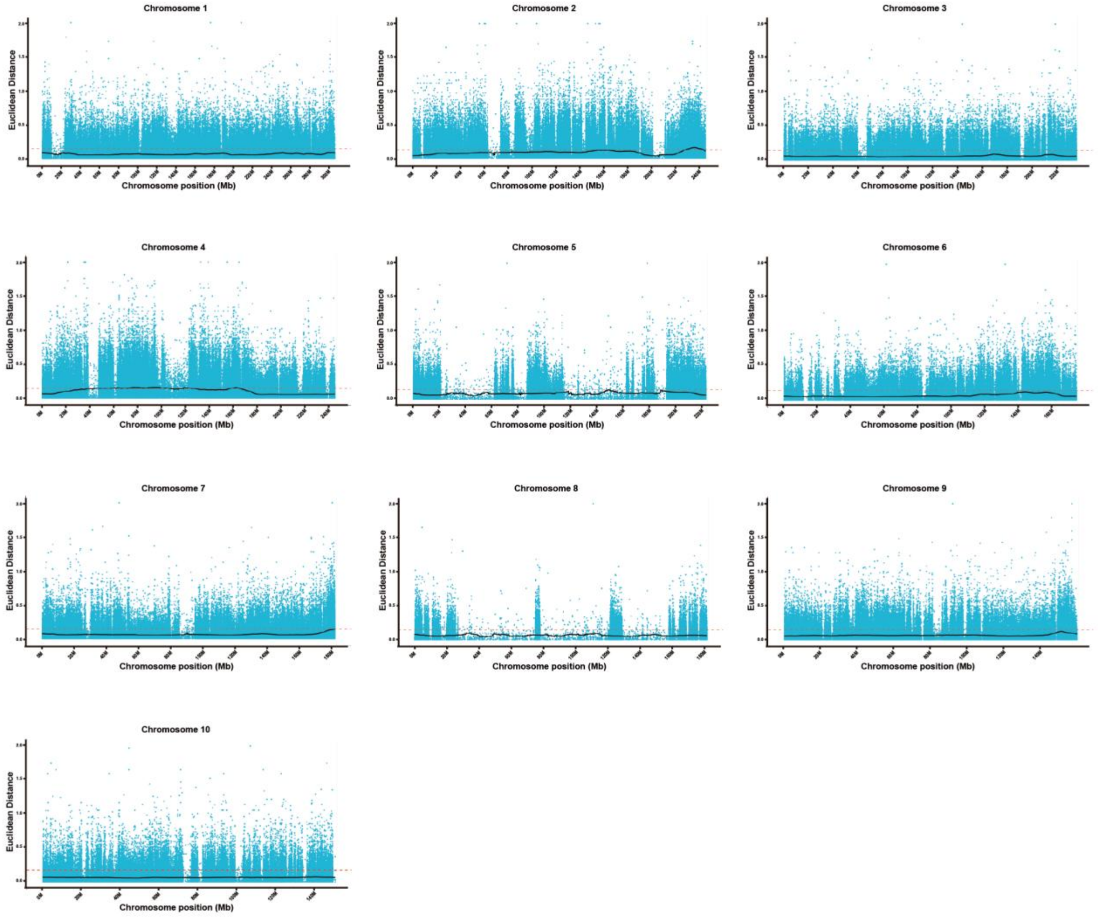

Figure S2 The distribution of ED_values for high-quality SNPs to the GER resistance on different chromosome. The ED value represents the size of the difference between the GER_S and GER_R DAN pools. The red dashed line represent the correlation threshold was calculated as 0.25.

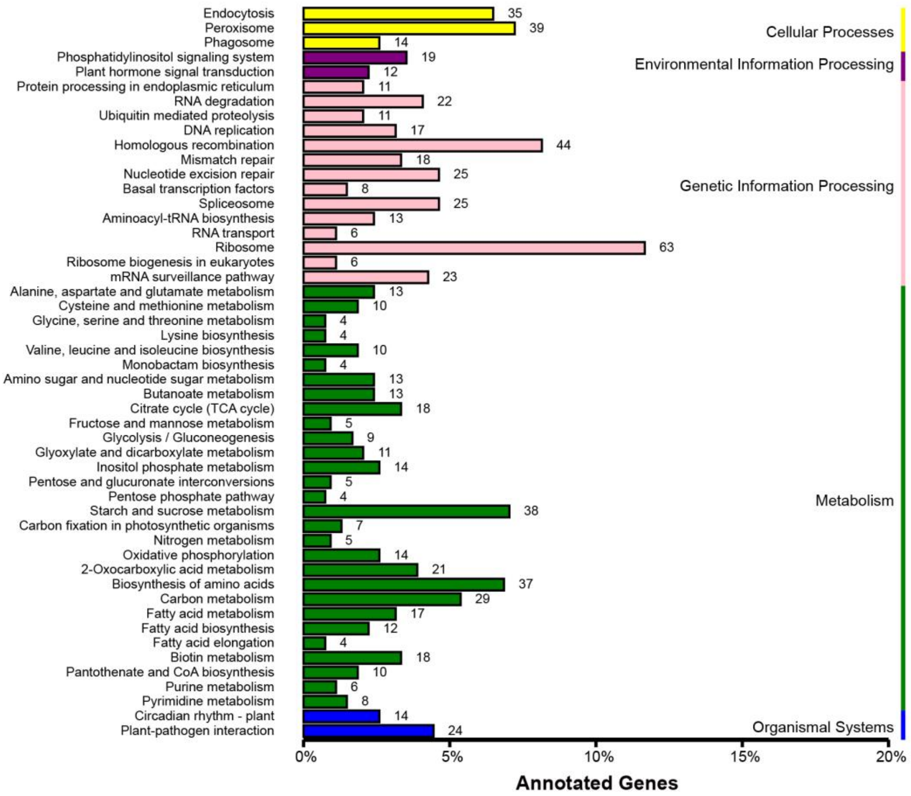

Figure S3 The KEGG_enrichment for whole genes in FER QTLs regions.

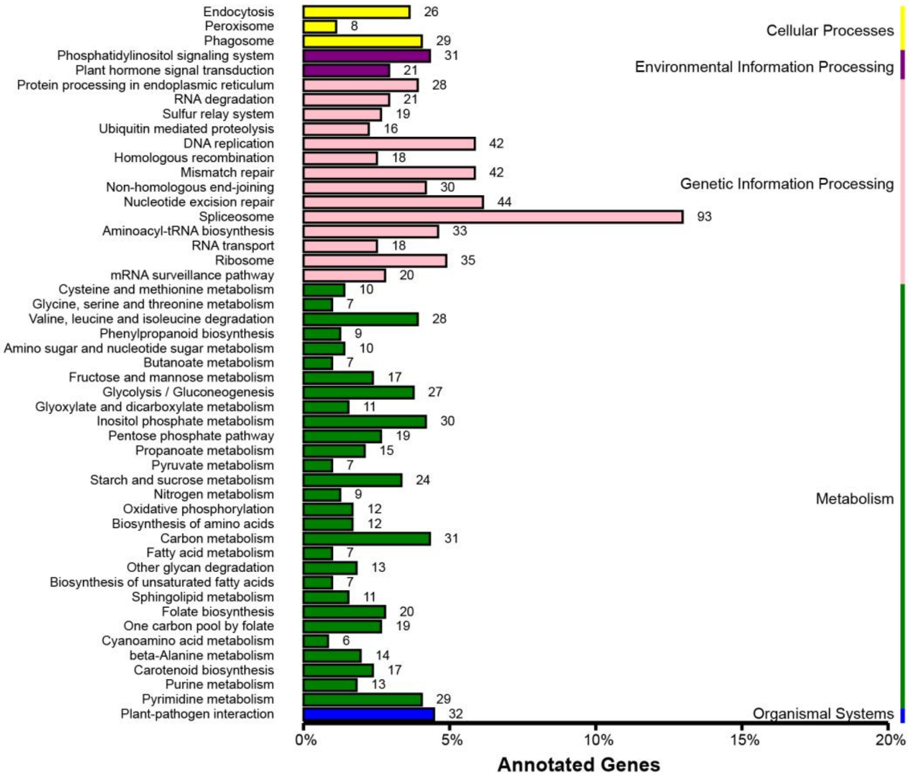

Figure S4 The KEGG_enrichment for whole genes in GER QTLs regions.
